# Awareness, knowledge, risk perception and uptake of maternal vaccination in rural communities of Ebonyi State, Nigeria

**DOI:** 10.4314/ahs.v22i4.36

**Published:** 2022-12

**Authors:** Ugochukwu Chinyem Madubueze, Alfred Friday Igwe Una, Ijeoma Nkem Okedo-Alex, Victor Maduka Agha, Chukwuma David Umeokonkwo, Irene Ifeyinwa Eze, Rowland Utulu, Kingsley Chijioke Okeke, Urudinachi Nnenne Agbo, Lilian Ndidiamaka Nwobashi, Chihurumnanya Alo, Benedict Ndubueze Azuogu

**Affiliations:** 1 Department of Community Medicine. Alex-Ekwueme Federal University Teaching Hospital Abakaliki (AE-FUTHA), Ebonyi State. Nigeria; 2 Community Medicine Department, Alex-Ekwueme Federal University Ndufu-Alike, Ikwo (AE-FUNAI), Ebonyi State. Nigeria; 3 Community Medicine Department, Ebonyi State University (EBSU); 4 African Institute for Health Policy and Health Systems, Ebonyi State University (EBSU), Nigeria; 5 Microbiology Department, Faculty of Science, Ebonyi State University (EBSU), Ebonyi State. Nigeria; 6 Department of Pediatrics, Alex-Ekwueme Federal University Teaching Hospital Abakaliki (AE-FUTHA), Ebonyi State. Nigeria

**Keywords:** Maternal vaccination, rural communities, Nigeria

## Abstract

**Introduction:**

Knowledge and uptake of maternal vaccination has been reported to be low in low- and middle-income countries.

**Objectives:**

To determine the knowledge, uptake and determinants of uptake of maternal vaccination among women of child-bearing age.

**Methods:**

A cross sectional study was done among 607 women of childbearing age selected from rural communities in Ebonyi State using multi-staged sampling technique. A pretested, interviewer administered questionnaire was used. The proportion of maternal vaccination uptake and predictors of uptake was determined at 5% level of significant using multiple logistic regression model.

**Results:**

Most of the respondents (39.9%) were in the 15–24 years age group. Only 1.3% and 41.5% were knowledgeable and had received any form of maternal vaccines respectively. The main reasons adduced for non-receipt of the vaccine was lack of information (65.8%) and not being pregnant (23.5%). Pregnancy was the predictor for uptake of maternal vaccine among the study population.

**Conclusions:**

There was low level of knowledge and uptake of maternal vaccine among rural women and a myth that the vaccine is only given when pregnant. This calls for increase targeted enlightenment of rural women on maternal vaccine in order to improve uptake.

## Background information

Maternal immunization is a cost-effective method of protecting mothers and children from some vaccine-preventable diseases which are significant causes of morbidity and mortality^1^. Approximately 1.5 million deaths in children under the age of 5 in 2018 were from vaccine-preventable diseases^2^. Immunization is thought to save at least 2–3 million lives each year with Hepatitis B vaccine alone responsible for another 600,000 lives saved 2,3.

Immunization of mothers during pregnancy has played a key role in lowering mortality in mothers and their offspring. The elimination of neonatal tetanus (NNT) for instance, leverages on maternal immunity obtained through vaccination in pregnancy 1. This has been a key component of the ‘Maternal and Neonatal Tetanus Elimination Initiative’ that contributed to the 96% decline in deaths from 787,000 recorded in 1988 to 30,848 in 2017.^1^,^4^

Maternal immunization with at least two doses of tetanus vaccine has been shown to reduce tetanus mortality by at least 94%^5^. The immunity conferred upon the mother from vaccination during pregnancy can be passed on to the child and provide some immunity during infancy albeit this is short-lived. 6 Passive immunity through trans-placental transfer of immunoglobulins from mother to fetus provides protection for infants for up to six months of life 6. In many low- and middle-income countries deliveries take place in less-than-optimal conditions and the risk of infections like neonatal tetanus and Hepatitis B is high 7.

Despite the proven effectiveness of maternal vaccination against vaccine-preventable diseases, uptake is still low in LMIC. Studies have identified knowledge as a key factor in determining vaccine uptake among mothers, with knowledgeable mothers being more likely to get vaccinated 8–10. Much of what is known about maternal vaccination in many LMIC like Nigeria occurs during antenatal clinics. 8–12 These clinics affords healthcare workers the opportunity to educate mothers on the benefits of vaccines against certain diseases. But with a significant proportion of mothers not attending antenatal clinics or delivering in hospitals knowledge of maternal vaccination and its uptake is suboptimal.^12^ Extensive research into factors that are responsible for non-vaccination in children exists but research into knowledge of maternal vaccination among mothers in countries like Nigeria is scant.^8^,^13^ However, available research seems to suggest poor knowledge of maternal vaccination and its ability to protect both mother and infant.^8^,^12^ Therefore, this study seeks to assess the awareness, knowledge, risk perception and uptake of maternal vaccination among women in rural communities in Ebonyi State Nigeria.

## Method

### Study Location

This study was conducted in rural areas in Ebonyi State. Ebonyi State is in the South-East geopolitical zone of the Federal Republic of Nigeria. The state is divided into 3 senatorial zones; Ebonyi North, Ebonyi Central and Ebonyi South with a total of 13 Local Government area (LGAs).^14^ The study was conducted in 3 communities, each selected from the senatorial zones.

### Study design, sampling technique and study population

This study is a cross sectional descriptive study targeted at women of reproductive age (15–49) in rural communities. The sample size for this study was calculated using the Cochrane formula n = Z^2^ pq/d^2^. The minimum sample size calculated was 629 with a p of 62%,^15^ and 10% non-response rate however 607 questionnaires were analysed.

A multi-staged sampling technique was used to select participants. The first stage was to select one local government area (LGA) each through simple random sampling from the 3 senatorial zones of Ebonyi State (Ebonyi North has 4 LGAs, Ebonyi Central has 4 LGAs and Ebonyi South has 5 LGAs). The second stage was to select one ward each from each selected LGA through simple random sampling after excluding the urban wards and subsequently one community each from the wards were selected. Only rural wards were included in this study. Households in the selected Communities were numbered and listed. Systematic random sampling was used to select the households in each community. In each household, one eligible female was selected by balloting where there was more than one eligible woman. The sampling continued until the desired sample size was reached. Only women of reproductive age (15–49 years) in the selected communities were included in this study and women who did not give consent to be part of this study were excluded.

### Data collection

Equal number of respondents (207) were interviewed per community. A pre-tested interviewer administered questionnaire was developed and used to collect information from the study participants. The questionnaire had four sections; which included socio-demographic characteristics, awareness and information need on maternal vaccination, knowledge of maternal vaccination schedule and respondents' risk perception and up-take of maternal vaccination. To validate the questionnaire used in this study the Cronbach alpha coefficient id was 0.78 and the questionnaire was pretested in a different community but with similar socio-demographic characteristics as the selected communities for this study. Data was collected in the local dialect of the respondents and incomplete data were termed invalid after data cleaning.

### Data Analysis

Measurement of awareness, source of information and the need for more information on maternal vaccination was assessed using proportion of 6 questions. Knowledge of maternal vaccination was measured with a set of nine stem questions with 17 possible correct responses. Each correct response was scored one and every wrong response was scored zero. There was therefore a maximum of 17 points. Which was converted to percentage. All respondents with greater than or equal to 50% was categorised as good knowledge, and a score less than 50% as poor knowledge. Uptake of maternal vaccine was defined as women of reproductive age who had ever received any maternal vaccination (TT and/or HBV).^16^ The risk perception was also assessed using proportions. Statistical Package for the Social Sciences (IBM-SPSS) for Microsoft Window version 20 was used for the data analysis. Frequency tables were used to present the descriptive statistics of the variables. Chi square statistics was used to examine the relationship between the uptake of maternal vaccine and other independent variables. Variable with p-value of 0.1 and less were inputted into a binary logistic regression model to identify determinants of maternal vaccination uptake.

### Ethical Considerations

Ethical approval EBSMOH/ERC/059/19 was obtained from the Health Research and Ethics Committee of Ebonyi State Ministry of Health. Written informed consent was obtained from the respondents prior to interview. Parental consent was obtained from those less than 18 after giving their accent.

## Results

Most of the respondents were in the age group 15–24 years (39.9%), had senior secondary education (39.7%), are farmers (28.8%) and 86.2% had their household income per month below N18,000 (Table 1). Majority of the respondents were married (63.6%) and mostly of monogamous type (52.2%). The respondents were predominantly Christians (99.7%) and of Roman Catholic denomination (71.2%). The reproductive history shows mean parity of 5.2±2.5. About 84.3% of the respondents had ever been pregnant, 13.3% are currently pregnant and 82.4% had desire for more children (Table 1).

**Table 1:** Sociodemographic and reproductive characteristics of respondents

Variables n=607	Frequency (%)
**Age group (years)**	
15–24	242 (39.9)
25–34	184 (30.3)
>34	181 (29.8)
Mean (SD)	27.8±8.7
**Occupation**	
Farmers	175 (28.8)
Petty traders	137 (22.7)
Unemployed	104 (17.1)
Artisan	101 (16.6)
Student	76 (12.5)
Civil servants	14 (2.3)
**Education**	
No formal education	49 (8.1)
Primary	194 (32.0)
Junior secondary	105 (17.2)
Senior secondary	241 (39.7)
Tertiary	18 (3.0)
**Marital status**	
Married	386 (63.6)
Single	192 (31.6)
Widowed	29 (4.8)
**Religion**	
Christianity	605 (99.7)
Traditionalist	2 (0.3)
**Type of marriage if ever married** **(n=415)**	
Monogamy	317 (52.2)
Polygamy	98 (16.1)
**Household income per month**	
<N18,000	523 (86.2)
N18,000 –N50,000	71 (11.6)
N51,000 –N100,000	7 (1.2)
N101,000 –N500,000	2 (0.3)
>N500,000	4 (0.7)
**Denomination**	
Catholic	432 (71.2)
Pentecostal	110 (18.1)
Presbyterian	54 (8.9)
Others	11(1.8)
**Ever been pregnant (n=607)**	
Yes	512 (84.3)
No	95 (15.7)
**Currently pregnant**	
Yes	190 (13.3)
No	417 (68.7)
**Couple desire for more children (n=415)**	
Yes	342 (82.4)
No	73 (17.6)
**Mean Parity (n=512)**	5.2±2.5
**Mean number of Children (n=512)**	4.7±2.3

Table 2 shows that 98.7% of the respondents have poor knowledge of maternal vaccination schedule. About 65.2% ever heard about vaccination of mothers, 50.2% ever heard about tetanus and 19.6% ever heard about hepatitis. The major source of information on immunization was health workers (68.1%) and 69.0% of the respondents also preferred healthcare workers as their source of information on immunization. Ninety-seven percent of the respondents stated desire for more information on vaccination.

**Table 2:** Overall knowledge, Awareness, sources and need for Information on maternal Vaccination

Variable (n=607)	Frequency (%)
**Knowledge grade**	
Poor knowledge (0–49)	599 (98.7)
Good knowledge (≥50)	8 (1.3)
**Ever heard about vaccination for mothers**	
Yes	396 (65.2)
No	211 (34.8)
**Ever heard of Hepatitis**	
Yes	119 (19.6)
No	488 (80.4)
**Ever heard about Tetanus**	
Yes	305 (50.2)
No	302 (49.8)
**Source of information(n=396)**	
Health worker	270 (68.1)
Friends and relatives	54 (13.6)
Religious leaders	32 (8.0)
Mass media	25 (6.3)
Roleplay/Drama	8 (2.0)
Social media	7 (2.0)
**Need more information about vaccination**	
Yes	589 (97.0)
No	18 (3.0)
**MOST preferred means for getting** **information on Immunization**	
Health workers consultation	419 (69.0)
Religious leaders	72 (11.9)
Through role play/drama	43 (7.1)
Mass media	32 (5.3)
Friends and relations	18 (2.9)
Social media	7 (1.2)
School	6 (1.0)
Others	10 (1.6)

Table 3 shows that 37.7% of the respondents correctly responded that tetanus toxoid is a routine immunization for women of reproductive age and 8.2% knew the correct number of doses needed for life-long immunity. Also, 12.9% correctly answered that Hepatitis B vaccine is a routine immunization for women of reproductive age and 5.9% knew the correct number of doses. Only 34.3% of the respondents knew that women get vaccinated to prevent vaccine preventable diseases in pregnancy while 29.5% and 13.3% knew vaccines protects their unborn child and prevents disability respectively. Only a few of the respondent knew the mode of transmission of hepatitis B; blood transfusion 12.2%, MTCT 4.%, via umbilical cord 2.6% while 66.7% responded that hepatitis could be transmitted via cough droplets. Only 20.4%, 14.3% and 9.3% knew open wound, piercing injuries and umbilical cord respectively were modes of transmission of tetanus. Only 39.0% of the respondents correctly responded that tetanus toxoid vaccination prevents diseases that causes muscle contraction and 19.6% knew that Hepatitis B vaccine prevents disease that affects the blood and Liver.

**Table 3:** Respondents' knowledge on maternal vaccination

Variable (n=607)	Frequency (%)
**Tetanus toxoid is a routine immunization for** **women of reproductive age**	
Correct response	229 (37.7)
Incorrect response	378 (62.3)
**Hepatitis B Vaccine is a routine immunization for** **women of reproductive age**	
Correct response	78 (12.9)
Incorrect response	529 (87.1)
**How many doses of Tetanus toxoid is needed for** **life long?**	
Correct response	50 (8.2)
Incorrect response	557 (91.8)
**How many times should a reproductive age** **woman receive the Hepatitis B**	
Correct response	36 (5.9)
Incorrect response	571 (94.1)
**Women get vaccinated to;**	
Prevent vaccine preventable diseases in pregnancy	208 (34.3)
Protect the unborn babies	179 (29.5)
Prevent from disabilities	87(13.3)
**Hepatitis can be transmitted through;**	
Blood Transfusion	74 (12.2)
Mother -to-child transmission	27 (4.4)
Via Umbilical cord	22 (3.6)
Sharing sharp objects	16 (2.6)
Cough droplets*	405 (66.7)
**Tetanus can be transmission through;**	
Open wound	124 (20.4)
Piercing injuries	87 (14.3)
Via Umbilical cord	60 (9.9)
MTCT	34 (5.6)
Blood and blood products*	56 (9.2)
Droplet infection*	6 (0.9)
**Tetanus toxoid vaccination prevents;**	
Diseases that cause muscle contraction	237 (39.0)
Disease that causes severe cough*	29 (4.8)
Disease that affects the blood and liver*	26 (4.3)
**Hepatitis B vaccine Prevents;**	
Disease that affects the blood and Liver	119 (19.6)
Disease that causes muscle contraction*	75 (12.4)
Disease that causes severe cough*	24 (3.9)

* Wrong responses

Table 4 shows that 41.5% of the respondents ever received any of the vaccines meant for mothers, about 41.4% of the respondents ever received TT with 71.7% receiving 1–4 doses and 13.5% receiving complete dosage for TT. Only 12.4% ever received HBV with 17.3% receiving 1–2 doses while only 25.3% had completed the schedule for HBV. The main condition for which vaccine was received was pregnancy (87.4%). About 65.8% of the respondents were yet to receive any of the vaccines because they did not know about some of the vaccines. Majority of the respondents (96.5%) stated willingness to vaccinate.

**Table 4:** Uptake, practice and risk perception towards vaccination among the respondents

Variable (n=607)	Frequency (%)
**Have you ever received any of the vaccines meant for mother?**	
Yes	252 (41.5)
No	355 (58.5)
**Ever received TT**	
Yes	251 (41.4)
No	356 (58.6)
**How many doses of Tetanus toxoid did you receive altogether?**	**n = 251**
1–4	180 (71.7)
5	34 (13.5)
More than 5	21 (8.4)
Not sure	16 (6.4)
**Ever received HBV**	
Yes	75 (12.4)
No	532 (87.6)
**How many doses of Hepatitis B Virus vaccine did you** **receive? (n=75)**	
1–2	13 (17.3)
3	19 (25.3)
Not sure	43 (57.3)
**What was the main condition for which you receive the vaccine?** **(n=326)**	
I was pregnant	285 (87.4)
I was of age as a woman for the vaccination	23 (7.1)
I was sick	7 (2.1)
Other reasons	11 (3.4)
**What was the MAIN reason why you are yet to receive any of the** **vaccines? (n=281)**	
I did not know about some of the vaccines	185 (65.8)
I was never pregnant	66 (23.5)
There was no need since I was never sick	15 (5.3)
Scarcity of vaccines	10 (3.6)
Distance to clinic	5 (1.8)
**Willingness to vaccinate (n=607)**	
Yes	586 (96.5)
No	21 (3.5)
**Do you feel vaccination is harmful**	
Ye	35 (5.8)
No	572 (94.2)
**Will you recommend vaccination to others**	
Yes	586 (96.5)
No	21 (3.5)
**How confident are you that you will get vaccinated**	
Strongly confident	393 (64.7)
Confident	165 (27.2)
Undecided	15 (2.5)
Rarely/never	34 (5.6)
**How confident are you to complete Tetanus toxoid vaccination**	
Strongly confident	393 (64.7)
Confident	165 (27.2)
Undecided	15 (2.5)
Rarely/never	34 (5.6)

The table also shows that 94.2% of respondents do not feel that vaccination is harmful and 96.5% will recommend vaccination to others. About 64.7% are strongly confident that they will get vaccinated and would complete tetanus toxoid vaccination.

Table 5 shows that factors significantly associated with uptake of maternal vaccine among the respondents were age, marital status, educational level, ever been pregnant and currently being married.

**Table 5:** Factors associated with uptake of maternal vaccine among the respondents

Variable	Maternal vaccination uptake		Chi square	P value
	Yes (%)	No (%)		
**Age (years)**				
15–24	40 (16.5)	202 (83.5)	103.927	<0.001
24–34	110 (59.8)	74 (40.2)		
> 34	102 (59.8)	79 (43.6)		
**Marital status**				
Married	224 (58.0)	162(42.0)	133.317	<0.001
Divorce/Separated/Widowed	13(44.8)	16(55.2)		
Single	15 (7.8)	177 (15)		
**Education**				
Primary or less	213 (42.4)	289(57.6)	1.000	<0.001
Secondary or more	39(37.1)	66 (62.9)		
**Ever been pregnant**				
Yes	203(39.6)	309 (60.4)	4.698	<0.001
No	49 (51.6)	46 (48.4)		
**Currently Pregnant**				
Yes	12 (6.3)	178 (93.7)	141.136	<0.001
No	240 (57.6)	177 (42.4)		

Table 6 shows that ever being pregnant and being currently pregnant are predictors of maternal vaccine uptake. Women who had ever been pregnant were 1.9 times more likely to take-up maternal vaccination compared to those that have never been pregnant, (aOR 1.9; 95%CI: 1.23–3.11). Likewise, respondents who were currently pregnant were 8.3 times more like to be vaccinated compared to those currently not pregnant (aOR:8.3;95%-CI:2.75–24.99).

**Table 6:** Determinants of maternal vaccine uptake among the respondents

Variable	AOR (95%CI)	P value
**Age (years)**		
15–24	1	
24–34	0.8 (0.45–1.55)	0.582
≥35	1.2 (0.83–1.95)	0.254
**Marital status**		
Married	1	
Not currently married	1.2 (0.33–4.55))	0.747
**Education**		
Primary or less	1	
Secondary or more	0.6 (0.34–1.09)	0.093
**Ever been pregnant**		
Yes	1.9 (1.23–3.11)	0.010*
No	1	
**Currently Pregnant**		
Yes	8.3 (2.75–24.99)	<0.001*
No	1	

* Statistically significant

## Discussion

This study assessed the awareness, knowledge, uptake, risk perception and determinants of uptake of maternal vaccination among women of reproductive age in rural communities in Southeast Nigeria.

Although majority (65%) were aware of maternal vaccination, we found that there was low awareness of maternal hepatitis B vaccination as only 19.6% had ever heard of this vaccine. In contrast, half of the respondents were aware of the tetanus toxoid (TT) vaccination for women. This could be because TT is routinely offered to pregnant women as a component of facility-based and non-facility-based antenatal care in the Nigerian context 17 and majority (84%) of the women had been pregnant in the past. This is further corroborated by the fact that the major and preferred source of information on maternal vaccination in this study was health workers. The fact almost half of the respondents had ever received TT possibly also enabled contact with health providers and information on TT by extension as this is a known content of health talks given to pregnant women during antenatal care.^8^,^11^,^18^ Health workers have been highlighted as the predominant source of information on maternal tetanus toxoid vaccination on previous studies.^19^,^20^ Women are more willing to receive vaccines if recommended by their health provider.^21^ Tetanus toxoid is also routinely offered to adults with puncture injuries even in patent medicine vendor settings and this could contribute to making it a more ‘popular’ vaccine than hepatitis B vaccine.

However, overall knowledge of maternal vaccines was quite suboptimal as 98.7% had poor knowledge. Critical areas of low knowledge were with respect to routinization and dosing of maternal vaccines, aims of maternal vaccination, transmission routes and affectation of these maternal vaccine-preventable diseases. This demonstrated low knowledge of maternal vaccines is particularly worrisome as low knowledge about maternal vaccines has been identified as a major barrier to vaccination acceptance during pregnancy.^22^ Poor maternal knowledge around vaccines is one of the fundamental mechanisms of vaccine hesitancy.^23^ Poor knowledge has also been identified as a barrier to maternal immunization uptake^24^ thus our findings further emphasize the need for targeted community-level interventions to improve awareness and knowledge as a precursor of uptake. In contrast with our findings, another study conducted in Lagos, Nigeria found a high level of awareness of tetanus immunization however, this study was carried out among urban residents and this could have affected their exposure to information on maternal vaccination. Despite the high level of awareness in the study, knowledge of tetanus toxoid dosing was also inadequate. 25 Another study among women of childbearing age in a tertiary institution in Osun, South-west Nigeria found that 64.5% of the women had poor knowledge of the causes of and risk factors for neonatal tetanus. The proportion of poor knowledge in that study is slightly better compared to our study which showed a much higher proportion (98.7%) of respondents with poor knowledge this may be because that study was conducted in an urban educational setting presumed to have more access and exposure to information. 19 Likewise, similar studies among Egyptian and Pakistani women found that majority of the respondents had poor knowledge of both maternal/neonatal tetanus and tetanus toxoid vaccine. 26,27

In contrast, a study in Zaria, Northern Nigeria found that the surveyed women were knowledgeable about neonatal tetanus and maternal immunization against tetanus. The preponderance of several institutions of learning in the study area and the relatively high educational status of the respondents were the major reasons accounting for this finding. 20

Slightly below half of the respondents had ever received a maternal vaccine likewise, individual vaccine uptake was even lower as only 41.4% had been vaccinated with TT. Similarly, other studies done in Pakistan and Ethiopia also found that only 43%-51.8% of women had received at least one dose of TT vaccinations.^27^–^29^ In an urban Nigerian study, only about 20% of the respondents had received two or more doses of the TT vaccine.^25^ In contrast, another clinic-based study among Egyptian women found that 60.6% had taken all required doses of TT vaccines however, because this was not a community-based study, its findings should be interpreted with caution when compared to the findings from our study.^30^

Although all women of childbearing age qualify to receive TT, 87.4% of our respondents received this vaccine while pregnant thus highlighting the need to increase awareness creation on uptake among non-pregnant women as this will facilitate the 5-dose completion for lifelong protection. This is also a call to optimize antenatal care attendance utilization among pregnant women in this context since this antenatal clinic has been shown to be an effective vehicle for the delivery of TT. 11 This is especially when antenatal care incorporates patient-centred education and interactive dialogues on maternal vaccination.^31^ Only 12.4% of the participants in this study had ever received Hepatitis B vaccine (HBV). Although vaccination with HBV is recommended for non-pregnant women who do not show evidence of infection and for pregnant women at risk for HBV infection during pregnancy, its uptake remains suboptimal.^32^ More clinic and community-based sensitization and emphasis is required to promote maternal immunization with HBV given its proven protection in the newborn and the high rates of chronic infection among neonates.^33^ Although these maternal vaccines are routinely provided to newborns in the national immunization schedule, prenatal maternal immunization remains highly recommended with the potential to protect pregnant women, fetuses and infants from several vaccine-preventable diseases.^33^,^34^

Poor knowledge of these maternal vaccines was the main reason (65.8%) cited by the respondents yet to receive the vaccines however most of the women did not consider vaccination harmful and were willing to receive and recommend maternal vaccination. Knowledge-related barriers to uptake of maternal vaccination has also been elucidated from other studies. 29 This further reinforces the need for community-level engagement with women and other stakeholders with regular risk communication and sensitization on maternal vaccination.

Pregnancy status (ever and currently being pregnant) was the predictor of uptake of maternal vaccinations identified in this study. Women who had ever been or were currently pregnant were more likely to have been vaccinated. This is understandable as our findings showed that most of the respondents received maternal vaccines especially TT while pregnant. Other studies have also found that women were more willing to get all recommended vaccinations if they have had at least one child.^35^ This willingness will invariably result in improved uptake of maternal vaccines. This further emphasizes the window of opportunity presented by the pregnancy state for maternal vaccinations. 11 Thus, more efforts should be targeted at ensuring that pregnant women access and complete the maternal vaccination schedules by utilizing integrated community and facility-based approaches aimed at optimizing ANC use and knowledge translation. Adequate provider basic and continuing education and capacity building on communication strategies techniques along this spectrum is also important. 36 Although the pregnant state should be optimized for maternal immunization, this finding also underscores the importance of proper community education and re-orientation on the need for such immunizations for women of childbearing status regardless of pregnancy status.

This study presents the following strengths. Firstly, it is one of the few studies in our context that presents evidence on both tetanus and hepatitis maternal vaccination from a large randomly selected sample of pregnant and non-pregnant women of childbearing age. Secondly, this study was community-based and was conducted in rural areas in contrast with facility-based urban studies.

The use of self-reports to assess uptake of maternal vaccination could have introduced social desirability bias however respondents were encouraged to give sincere responses and were assured of the confidentiality of the responses. Our findings may also have limited generalizability to urban settings.

## Conclusion

This study found low levels of awareness and knowledge of maternal vaccination with high willingness to be vaccinated among the respondents. There were suboptimal levels of uptake of tetanus toxoid vaccine. Only a small proportion of the participants had been vaccinated against hepatitis B. Ever and currently being pregnant were the predictors of maternal vaccination. We recommend sustained implementation of both community and facility-based awareness creation on maternal vaccination aimed at improving knowledge and uptake among women of childbearing age regardless of pregnancy status. Existing community women social groups such as religious, kindred and occupational associations can serve as platforms for targeted enlightenment on maternal vaccines among women in rural areas.
